# Blood Lead Levels Among Pregnant Women: Historical *Versus* Contemporaneous Exposures

**DOI:** 10.3390/ijerph7041508

**Published:** 2010-04-01

**Authors:** Marie Lynn Miranda, Sharon E. Edwards, Geeta K. Swamy, Christopher J. Paul, Brian Neelon

**Affiliations:** 1 Nicholas School of the Environment, Duke University, Box 90328, Durham, NC 27708, USA; E-Mails: se@duke.edu (S.E.E.); cjp2@duke.edu (C.J.P.); neelo003@duke.edu (B.N.); 2 Department of Obstetrics and Gynecology, Duke University Medical Center, 2608 Erwin Rd, Suite 200 Durham, NC 27705, USA; E-Mail: swamy002@mc.duke.edu

**Keywords:** blood lead, pregnancy, birth outcomes, lead exposure

## Abstract

Blood lead among pregnant women, even at modest levels, may impair offspring cognitive development. We examine whether blood lead levels (BLLs) result from current *versus* historic exposures, among a cohort of pregnant women. Cumulative logit models were used to characterize the relationship between maternal risk factors and higher BLLs. Maternal blood lead levels more likely result from lead remobilization from historic *versus* contemporaneous exposures. Even if all lead sources were abated immediately, women and their fetuses would experience lead exposure for decades. This work emphasizes the importance of addressing sources of environmental lead exposure in the United States and internationally.

## 1. Introduction

Medical professionals have long recognized severe lead poisoning as a debilitating disease. Since the late 1970s, however, mounting research demonstrates that lead causes irreversible, asymptomatic effects at levels far below thresholds previously considered safe. Research suggests that significant adverse health effects occur at blood lead levels (BLLs) below the current CDC blood lead action level of 10 μg/dL, such as learning and behavioral deficits [1–5] and decreased performance on standardized IQ and educational achievement tests [2–10]. Meta-analysis and reviews suggest that there is no threshold effect level, and thus any level of exposure is potentially detrimental [11–14]. A number of studies demonstrate that significant damage occurs even at blood lead levels below 5 μg/dL [5,9,15].

Children exposed to lead *in utero* and in the postnatal period tend to be smaller, weaker, less coordinated, and less intelligent than children who have not had significant exposure [2,16–19]. Lead exposure during the critical initial neurological development of a fetus may be particularly harmful [20]. Prenatal lead exposure has also been associated with criminality [21]. Prenatal exposure can occur if the mother is exposed to lead through infrastructure (*i.e*., lead-based paint), diet, cosmetics, or occupational hazards, or if she has lead stores in her body from previous exposure. Pregnant women and nursing mothers with high blood lead levels may experience elevations in both systolic and diastolic blood pressure [22,23]. In turn, elevated maternal blood pressure during pregnancy has been linked to adverse maternal health, as well as slowed and perturbed fetal development [24,25].

Blood lead levels in women of child-bearing age have been decreasing over the previous decades, but remain a concern. NHANES data from 1999 to 2002 suggest that women aged 20–59 nationally have a mean level of 1.2 μg/dL and 0.3% of women have blood lead levels above 10 μg/dL [26]. The rate is slightly lower for all females between age 6 and 19, at 0.2% with BLLs above 10 μg/dL and a mean BLL of 1.0 μg/dL. Non-Hispanic black women aged 20–59 have a significantly higher mean level of 1.4 μg/dL [26]. Other demographic predictors are consistent with those for children’s BLLs, including education, income, age of housing, as well as smoking, alcohol consumption, and living in an urban area [27].

Relatively little is known about blood lead levels in pregnant women. Rothenberg *et al*. examined the pattern of blood lead levels over the course of a pregnancy among 105 women residing in Mexico [28]. The authors documented a significant decrease in blood lead levels from week 12 to week 20, which they attribute to hemodilution and organ growth [28]. After 20 weeks, however, blood lead levels rose again, peaking at parturition. Similarly, Hertz-Piccioto *et al*. performed a nested cohort study of pregnant women in Pittsburgh, PA to examine the pattern of maternal BLLs over the course of gestation [29]. They identified the same distinct U-shaped pattern in BLLs as was found in the Rothenberg *et al*. study. Furthermore, they determined that older mothers had a steeper increase in BLLs during the latter half of pregnancy than did younger mothers, which was modified by calcium intake. Indeed, calcium supplementation may be a cost effective way to reduce maternal blood lead levels and thus fetal exposure [30]. The U-shaped pattern of blood lead remains despite supplementation, and Lamadrid-Figueroa *et al*. suggest that exposure is underestimated when plasma lead levels are not considered [31].

Positive findings of blood lead among pregnant women may be indicative of contemporaneous exposure to lead or may result from remobilization of lead from bone stores due to either the aging process or the physiological stress of pregnancy. This paper has two purposes: first, to document the blood lead burdens among a cohort of pregnant women in Durham County, NC; and second, to model predictors of blood lead level, with a particular emphasis on disentangling current *versus* historic exposures. The results of the analysis are directly relevant to state, national, and international lead policies, as well as the aggressiveness with which the policies should be pursued.

## 2. Data and Methods

The Healthy Pregnancy, Healthy Baby study is an ongoing prospective cohort study of the effects of environmental, social, and host factors on racial disparities in pregnancy outcomes. Duke University Medical Center (DUMC) Institutional Review Board approval was obtained to enroll pregnant women from the Duke Obstetrics Clinic and the Durham County Health Department Prenatal Clinic. Women were excluded from participation if they were less than 18 years of age, were not English-literate, were greater than 28 weeks’ gestation at study enrollment, lived outside of Durham County, had a multi-fetal gestation, had a known fetal genetic or congenital abnormality, or were planning not to deliver at DUMC. Demographic, health behavior, and medical history data were obtained by direct patient interview at the time of enrollment and through electronic medical record review. Participants recruited from June 2005 to December 2008 are included in this analysis. Of the 1505 women approached for participation in the study, 1294 consented (86%). At the time of this analysis, we had lead levels available for 927 of the enrolled and consented women.

All participants were geocoded to the individual tax parcel unit based on the residential address reported at time of enrollment (96.7% georeferenced). Such highly resolved spatial referencing of the data allowed each participant to be linked with parcel level data on age of housing and lead exposure risk level. The age of housing (year built) for each parcel was provided by the Durham County Tax Assessor. Each tax parcel’s lead exposure risk level was calculated using a model of lead risk that has been validated by collection of environmental samples in homes in Durham County. Using tax assessor, lead screening, and U.S. Census data, a modeled lead exposure risk estimate for each residential tax parcel was calculated by weighting risk factors for lead exposure, including age of housing, Census blockgroup median income, and Census blockgroup percent African American (detailed methods described previously in [32,33]).

Maternal blood lead levels were measured in blood samples collected at the time of admission to the DUMC Birthing Center for delivery. Whole blood, collected in a trace-metal free vacutainer tube, was sent to the Mayo Medical Laboratories for graphic furnace atomic absorption spectrometry for determination of BLLs, with a lower limit of detection of 1.0 μg/dL [34].

Descriptive statistics were calculated to provide a sense of the cohort under study in terms of both demographic data and lead exposure. The population for this analysis was restricted to non-Hispanic white, non-Hispanic black, and Hispanic participants who completed the study and had a valid lead result available. As only 10.4% of women in our study had blood lead levels greater than 1 μg/dL, we categorized blood lead into three ordered groups: below the detection limit, 1 μg/dL, and ≥2 μg/dL. We then used cumulative logit models with a proportional odds assumption to assess the relationship between maternal risk factors and higher lead levels. The proportional odds assumption implies that the covariate effects are the same across logits and was tested *via* a score test. The score tests on all three models that we estimate support the validity of the proportional odds assumption. We report the results in terms of adjusted odds ratios, confidence intervals, and p-values. Because these analyses were exploratory in nature, tests of statistical significance did not include an alpha adjustment. All analyses were undertaken using SAS 9.2 (Cary, NC).

## 3. Results

Of the 1294 participants screened and enrolled between June 2005 and December 2008, 6.5% were lost to follow-up and 2.2% withdrew. Lead results were only available for participants completing the study because blood is collected for lead analysis at delivery rather than enrollment. Lead results were available for 927 participants at the time of this study. In addition, only non-Hispanic white, non-Hispanic black, and Hispanic participants were included in the analyses presented here. Under these restrictions, data on 864 participants were used in the overall analysis. Although almost 97% of participants were geocoded, age of housing data was available for 770 of these participants and modeled lead exposure risk was available for 701 of these participants.

Table 1 provides demographic characteristics of the study population for each subset. The demographic composition of the each subsets used for the models with a measure of current lead exposure (last two columns of Table 1) were not statistically different from the demographic composition of the participants in the basic model (p > 0.05 for χ^2^ tests of each categorical demographic variable; p > 0.05 for *t*-tests for differences in mean parity). Non-Hispanic black women account for almost three-fourths (72.6% in basic model) of the study population (oversampling intentional), and roughly two-thirds (65% in the basic model) of participants were under 30 years old. Parity measures the total number of deliveries to the mother, including previous term births, previous preterm births, and the current delivery. From both national and North Carolina birth data, we know that rates of tobacco use during pregnancy are typically highest among non-Hispanic white women and lowest among Hispanic women [35,36]. Our study population does not follow this typical pattern, with tobacco use rates highest among non-Hispanic black participants (data not shown here).

**Figure 1.**
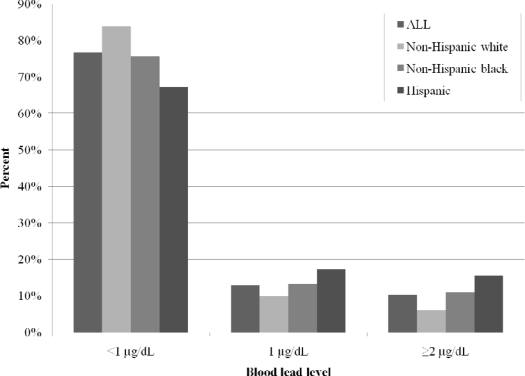
Blood lead levels by race.

Figure 1 displays the race-specific distributions of blood lead levels among the 864 women included in the basic model. As expected, the non-Hispanic white women in our sample generally had lower blood lead levels than non-Hispanic black and Hispanic women. In our study, Hispanic women had the highest blood lead levels, with 16% having ≥ 2 μg/dL compared to 6% among non-Hispanic white and 11% among non-Hispanic black women. Almost half of the Hispanic participants in our sample were not born in the United States, so maternal lead levels for these women may be related to non-US exposures to lead.

As shown in Table 1 and Figure 1, both overall and within each race, the vast majority of participants had blood lead levels below the detection limit of 1 μg/dL. Only 2 participants had a blood lead level of 5 μg/dL or higher. Due to this sparseness, lead levels over 1 μg/dL were collapsed into a single category, and blood lead levels were modeled as three ordered categories of <1 μg/dL, 1 μg/dL, and ≥2 μg/dL.

Three cumulative logit models for the ordered lead level categories were fit: (1) the basic model including race, age, education, parity, and tobacco use during pregnancy; (2) the basic model covariates plus age of housing; and (3) the basic model covariates plus modeled lead exposure risk. The categories of non-Hispanic white and 25–29 years of age served as reference groups. The score tests in all three models supported the assumption of proportional odds (score test χ^2^ = 5.39, p = 0.86 for basic model; χ^2^ = 4.97, p = 0.93 for age of housing model; and χ^2^ = 6.14, p = 0.86 for modeled exposure risk model), indicating that the cumulative logit models could be appropriately applied in this case.

Table 2 presents the results for each model. The adjusted odds ratios (aORs) presented in this table indicate the probability of having a higher blood lead level compared to the associated reference group. In all three models, race, age, and educational attainment were significantly associated with blood lead level (p < 0.05). Tobacco use during pregnancy was also significant in the basic and age of housing models (p < 0.05; p = 0.07 in the modeled exposure risk model). Non-Hispanic black and Hispanic race were significantly associated with increased blood lead level compared to non-Hispanic white race (aOR, basic model = 2.90 and 4.92; aOR, age of housing model = 3.49 and 4.54; and aOR, modeled exposure risk model = 3.11 and 3.84, respectively; all p < 0.05). Having less than a high school education and using tobacco during pregnancy were also associated with higher blood lead levels in each model (p < 0.05 in basic and age of housing models). Parity was not significantly associated with blood lead level in any of the models, but the adjusted odds ratios on parity were in the direction we would expect; *i.e*., women with previous pregnancies have lower blood lead levels, potentially indicating that maternal lead body burdens may have been at least partially off-loaded during the earlier pregnancies.

The probability of having a higher blood lead level significantly increased with age. In the basic model, compared to the referent of 25–29 years, participants aged 18–19 had an aOR of 0.60 (95% CI = 0.28, 1.27), those aged 20–24 years an aOR of 0.54 (95% CI = 0.33, 0.89), those aged 30–34 years an aOR of 2.39 (95% CI = 1.47, 3.91), those aged 35–39 years an aOR of 2.98 (95% CI = 1.71, 5.18), and those aged 40–44 years an aOR of 7.69 (95% CI = 3.49, 16.93). A similar pattern by age was found in both models that included a measure of current lead exposure; although in the third model which added modeled lead exposure risk, the 20–24 years age category was not significantly different (p = 0.058) from the 25–29 years referent group. Note, however, that age of housing was not significant in the second model and modeled lead exposure risk was not significant in the third model. Note also that the confidence intervals are wide in some cases due to low sample sizes.

In addition, we checked the sensitivity of the results to both outliers and the classification of blood lead level. The two participants with blood lead levels >5 μg/dL, who were originally included in all three models, were removed from the dataset and the set of cumulative logit models rerun. Removing these outliers did not influence the results or their interpretation. Fitting the three models as simple logistic models with blood lead levels dichotomized as detectable/non-detectable (<1 μg/dL and ≥1 μg/dL) also did not affect the results. All standard covariates followed similar patterns, both in terms of direction and statistical significance, as in the corresponding cumulative logit models. Again, the two measures of current lead exposure, age of housing and modeled lead exposure risk, were not significantly associated with blood lead level.

## 4. Discussion

Maternal blood lead levels can result from either contemporaneous exposure to lead or through the remobilization of lead that has been sequestered in mineralized tissue in response to a historic exposure. When distributed to mineralized tissue, especially bone, lead is mistaken for calcium and used as faulty building blocks [37]. An estimated 95% of adult’s lead burden is in their bones, compared to over 70% of child lead stores [38]. Women are considered be at greater risk than men for the remobilization, and much literature documents the significant disparity in the large lead burdens rates of blacks [26,39].

Research indicates that bone is a living organ that accumulates lead in three compartments with three different half-lives and continually remobilizes lead to the bloodstream and other organs. The first compartment, periosteum, resembles soft tissue and is very common in growing infants. Periosteum readily releases lead stores into the bloodstream [38]. A second compartment is the spongy or trabecular bone (found in the pelvis, ribs, and skull) with a half-life of 3–5 years. The third compartment is cortical bone (midtibia and midfemur) and has the longest resident time with a half-life of about 30 years [40].

Maternal blood lead levels are inextricably tied to biological processes associated with calcium needs, absorption, and desorption. Pregnancy clearly induces a demand for calcium within the fetal compartment. Maternal responses to meet this demand can occur through increased absorption of calcium in the intestinal tract, changes in calcium conservation *via* kidney function, or calcium desorption from bone [41]. If lead has been absorbed into bone, then a remobilization process may be induced by pregnancy itself, due to the demand for calcium within the fetal compartment.

Maternal blood lead levels may be related to the aging process as well. Peak bone mass is generally considered to be achieved in young adulthood [42]. After bone mass peaks, bone loss begins, with resorption greater than formation [42]. Again, if lead has been used as a faulty building block in place of calcium, then we would expect blood lead levels to increase as women age. In our analysis, maternal age was strongly associated with blood lead levels, with younger women having a reduced risk and older women having an elevated risk. This result may be indicative of the resorption of bone lead in pregnancy. It may also be related to the fact that, due to the temporal patterns of lead exposure, older mothers (compared to younger mothers) may have been exposed to more lead on average during their childhoods. Other research has suggested a strong connection between bone lead and health outcomes potentially arising from exposure. In one study, bone lead predictions had more significant associations with hypertension than blood lead [43].

This study is limited to women from one county in North Carolina in a study population that intentionally oversamples non-Hispanic black women. As such, it should be replicated in other study populations to confirm the results found here. In addition, we are unable to incorporate direct measures of bone turnover, nutritional intake, or genetic vulnerabilities, all of which may contribute to blood lead levels among pregnant women. Nevertheless, we feel that the results presented here constitute an important contribution to the literature on lead levels during pregnancy.

Maternal blood lead levels may also be related to current exposures. In our analyses, however, neither measure of current lead exposure (age of housing and modeled lead exposure risk) was significantly associated with blood lead levels. The aOR for age of housing was 1.00 (95% CI = 0.99, 1.01) and for modeled lead exposure risk was 0.88 (95% CI = 0.18, 4.29). Taken in combination with the results on maternal age, this finding indicates that maternal blood lead levels are much more likely the result of lead remobilization from historic exposures as opposed to contemporaneous exposures.

## 5. Conclusions

The 2005–2006 National Health and Nutrition Examination Survey (NHANES) data reveal blood lead levels elevated above the CDC action level of 10 μg/dL in 1.3 percent of one to five year olds in the United States, with children tested having an overall geometric mean blood lead level of 1.7 μg/dL [44]. These data indicate that over 500,000 children under age six currently experience blood lead levels above the CDC blood lead action level [45]. Given the recent research on health effects of low level lead exposure, roughly 37% of children aged one to five in the United States are estimated to have blood lead levels greater than or equal to 2 μg/dL [44].

The results from this analysis indicate that pregnant women themselves (and adults more generally) are likely serving as a reservoir for lead stores. These stores are remobilized to the blood stream through natural aging processes, and rates of remobilization are likely accelerated by the physiological stress and calcium homeostasis of pregnancy. Lead diffuses from the mother’s bloodstream to the fetus across the placenta and accumulates in fetal organs. From a public health perspective, this means that even if all lead sources were abated immediately and completely, women and their growing fetuses would remain at risk for lead exposure for decades. Yet a significant number of US children today still carry unacceptable blood lead levels. These numbers are even higher globally when considering the several countries that have limited or no restrictions on lead in gasoline and industry. Thus, this work again emphasizes the critical importance of aggressively addressing sources of environmental lead exposure both in the United States and internationally.

## Figures and Tables

**Table 1.** Demographic distribution of observations in each model.

	**Basic model**			**Basic model + age of housing**			**Basic model + modeled exposure risk**		
	**n**	**%**		**n**	**%**		**n**	**%**	
**Race**									
Non-Hispanic white	179	20.7%		159	20.7%		130	18.5%	
Non-Hispanic black	627	72.6%		563	73.1%		525	74.9%	
Hispanic	58	6.7%		48	6.2%		46	6.6%	

**Age (yrs)**									
18–19	70	8.1%		61	7.9%		57	8.1%	
20–24	301	34.8%		268	34.8%		251	35.8%	
25–29	191	22.1%		170	22.1%		159	22.7%	

30–34	166	19.2%		148	19.2%		132	18.8%	
35–39	107	12.4%		97	12.6%		81	11.6%	
40–44	29	3.4%		26	3.4%		21	3.0%	

**Education**									
Less than high school	111	12.9%		97	12.6%		91	13.0%	

**Parity**									
Mean			2.14			2.17			2.16
SD			1.27			1.29			1.25

**Tobacco use**	150	17.4%		128	16.6%		117	16.7%	

**Blood lead level**									
< 1 μg/dL	663	76.7%		583	75.7%		531	75.8%	
1 μg/dL	112	13.0%		105	13.6%		95	13.6%	
≥ 2 μg/dL	89	10.3%		82	10.7%		75	10.7%	

**Table 2.** Adjusted odds ratios and 95% confidence intervals for blood lead levels in cumulative logit models.

	**Basic model (n = 864)**		**Basic model + age of housing (n = 770)**		**Basic model + modeled exposure risk (n = 701)**	
	**aOR (95% CI)**	**P-value**	**aOR (95% CI)**	**P-value**	**aOR (95% CI)**	**P-value**
**Race**						
Non-Hispanic white	1.0 —	—	1.0 —	—	1.0 —	—
Non-Hispanic black	2.90 (1.79–4.70)	< 0.001	3.49 (2.09–5.85)	< 0.001	3.11 (1.75–5.52)	0.002
Hispanic	4.92 (2.39–10.09)	< 0.001	4.54 (2.04–10.13)	< 0.001	3.84 (1.65–8.89)	< 0.001

**Age (yrs)**						
18–19	0.60 (0.28–1.27)	0.179	0.57 (0.26–1.25)	0.160	0.65 (0.29–1.47)	0.296
20–24	0.54 (0.33–0.89)	0.015	0.51 (0.31–0.87)	0.012	0.60 (0.35–1.02)	0.058
25–29	1.0 —	—	1.0 —	—	1.0 —	—
30–34	2.39 (1.47–3.91)	< 0.001	2.47 (1.48–4.12)	< 0.001	2.50 (1.46–4.25)	< 0.001
35–39	2.98 (1.71–5.18)	< 0.001	3.32 (1.85–5.96)	< 0.001	3.25 (1.74–6.09)	< 0.001

40–44	7.69 (3.49–16.93)	< 0.001	6.27 (2.71–14.55)	< 0.001	6.83 (2.72–17.11)	< 0.001

**Education**						
Less than high school	2.17 (1.34–3.51)	0.002	1.99 (1.19–3.31)	0.008	2.07 (1.22–3.49)	0.007

**Parity**	0.90 (0.78–1.03)	0.118	0.863 (0.75–0.99)	0.045	0.91 (0.78–1.07)	0.265

**Tobacco use**	1.64 (1.07–2.50)	0.022	1.70 (1.08–2.66)	0.021	1.54 (0.96–2.48)	0.073

**Exposure measures**						
Age of housing (year built)			0.99 (0.98–1.00)	0.249		
Modeled lead exposure risk					0.99 (0.29–3.40)	0.986
